# Access to care and worsening eating disorder symptomatology in youth during the COVID-19 pandemic

**DOI:** 10.1186/s40337-021-00421-9

**Published:** 2021-06-10

**Authors:** Rebecca Spigel, Jessica A. Lin, Carly E. Milliren, Melissa Freizinger, Julia A. Vitagliano, Elizabeth R. Woods, Sara F. Forman, Tracy K. Richmond

**Affiliations:** 1 Division of Adolescent and Young Adult Medicine, Boston Children’s Hospital, 300 Longwood Ave, Boston, MA 02115 USA; 2 Department of Pediatrics, Harvard Medical School, 25 Shattuck Street, Boston, MA 02115 USA; 3 Institutional Centers for Clinical and Translational Research, Boston Children’s Hospital, 300 Longwood Ave, Boston, MA 02115 USA; 4 Department of Psychiatry, Harvard Medical School, 25 Shattuck Street, Boston, MA 02115 USA

**Keywords:** COVID-19, Eating disorders, Adolescents, Access, Telemedicine

## Abstract

**Background:**

Shelter-in-place orders and social distancing guidelines, in response to the COVID-19 pandemic, have limited traditional face-to-face interactions and led to many clinical providers transitioning to the use of videoconferencing platforms. The present study aims to assess how the COVID-19 pandemic has impacted adolescents’/young adults’ (AYA) eating disorder (ED)-related care, and how access to, changes in, perceived disruptions to, and quality of care are associated with ED thoughts and behaviors.

**Methods:**

AYA enrolled in the RECOVERY study, a pre-existing web-based longitudinal study, and completed a COVID-19-specific survey (*n* = 89). We examined bivariate associations of four markers of care: i) access to care, ii) changes in care, iii) perceived disruption to care, and iv) quality of care. Using multiple logistic regression, we examined the associations of pandemic-related markers of care with changes in ED thoughts and behaviors. We excluded those not engaged in treatment pre-pandemic (*n* = 16).

**Results:**

In the remaining 73 participants, reported access to care was high, with 92% of respondents continuing care with at least one ED provider during the pandemic; however, 47% stopped *some* treatment during the pandemic. Nearly one-third (32%) perceived a disruption in treatment. Quality of care remained high with 67% reporting care to be better than or as good as pre-pandemic. Respondents acknowledged heightened symptomatology: 81% reported increased ED thoughts and 81% reported increased ED behaviors due to COVID-19-related factors. However, none of the markers of care described were significantly associated with ED thoughts or behaviors in regression analyses adjusting for demographic variables and baseline characteristics, except our quality of care measure which was approaching significance (*p* = 0.07).

**Conclusions:**

Our findings show the majority of AYA who had care prior to the pandemic continued receiving some element of their multi-disciplinary ED treatment and perceived their care as high quality. None of the markers of care described were statistically associated with increased ED thoughts and behaviors.

## Plain ENGLISH summary

The COVID-19 pandemic has had a negative impact on our global community’s mental health, in particular those struggling with psychiatric illnesses, such as eating disorders (ED). Stay-at-home orders and social distancing have limited in-person access to ED treatment and as a result, many care providers have transitioned to using videoconferencing platforms. Clinicians who care for patients with EDs worry that these sudden changes in accessing treatment, on top of mental health challenges associated with the pandemic, may contribute to worsening ED symptoms. In this study, we asked adolescent and young adult patients with EDs about their symptoms, access to treatment, changes in care, disruptions in treatment and quality of care since the pandemic started. Our results demonstrate that patients with established care teams have maintained treatment and perceive their care as high quality, though the majority are experiencing worsening ED thoughts and behaviors. Patients who perceive their quality of care as worse than usual might be more likely to have intrusive ED thoughts. Continued access to care could also be protective against increased ED behaviors. Ultimately, our study highlights the need for continued support of patients during this challenging time.

## Background

Since the beginning of 2020, the COVID-19 pandemic has had a profound impact on youth around the world. Though these effects have been universal, individuals with pre-existing psychiatric illnesses are especially vulnerable to the consequences of COVID-19 [1]. Individuals with eating disorders (ED) are at particular risk, as stress and anxiety may lead to worsening eating disordered cognitions, which may further lead to negative behaviors and detrimental physical effects [2]. Not surprisingly, individuals with EDs have reported increased social isolation, rumination about eating, feelings of anxiety and depression, and decreased feelings of control and social support during the COVID-19 pandemic [3–5]. Clinicians working with patients with EDs have also voiced concern that the changes caused by COVID-19 may increase ED symptoms, decrease protective factors, and exacerbate barriers to care [6].

The standard of care for ED treatment involves a multi-disciplinary team to address the medical, psychological, and nutritional components of this serious, life-threatening psychiatric illness [7]. Shelter-in-place orders and social distancing guidelines have limited face-to-face interactions, leading to residential and day program closures and limiting in-person outpatient care from all disciplines. Thus, for most patients there have been reductions in in-person contact with clinical teams [8]. Recognizing the need to limit in-person clinical care, governing bodies responded by changing national and state payment policies and lifting technology restrictions, encouraging clinicians to maintain treatment continuity [9]. In response, providers across disciplines have rapidly transitioned their practices to videoconferencing platforms, commonly referred to as telehealth [10, 11]. ED clinicians have published recommendations on how to adapt multi-disciplinary ED care to telehealth [12–14], however, little is known about how patients perceive this change in their care as a result of the COVID-19 pandemic.

Experts worry that these sudden changes and limited access to in-person care, coupled with increasing mental health challenges associated with the pandemic, may have serious implications for patients with EDs [3]. The absence of in-person care creates specific challenges for ED treatment, namely, monitoring for changes in weight and vital signs, essential markers of illness [14, 15]. Additionally, some believe that the therapeutic alliance, necessary for successful clinical care, could be disrupted by the transition to telehealth [16]. Though many raise concerns that reduction in in-person access to clinical providers may contribute to the worsening of ED symptoms, little research currently exists [8, 12, 13, 17]. Thus, we set out to do the following: 1) examine four markers of care during the time of the COVID-19 pandemic: i) reported access to (telehealth and/or in-person), ii) changes to, iii) perceived disruption of, and iv) reported quality of ED-related care; and 2) examine whether these four markers of care are associated with increased ED thoughts and behaviors. We hypothesized that decreased access to, changes in, perceived lower quality of, as well as perceived disruption to care would be associated with increased ED thoughts and behaviors.

## Methods

### Study sample

We used data from the Registry of Eating Disorders and their Co-Morbidities Over Time in Youth (RECOVERY), a longitudinal registry of adolescent/young adult patients ages 10–27 (average age 17.1 years at enrollment) seeking ED treatment. RECOVERY study participants were previously recruited from the outpatient ED program at Boston Children’s Hospital from June 2017 to February 2020. Participants provided written consent and assent prior to enrollment. Using web-based surveys sent via Research Electronic Data Capture (REDCap a HIPAA compliant database) patients, their parents, and their clinicians answered questions regarding ED behaviors and treatment every 3 months for the first year and every 6 months thereafter. Participants received remuneration for each completed survey. In July 2020, in response to the COVID-19 pandemic, we invited all patient participants to complete an added survey with the aim of understanding the impact of the pandemic on ED treatment, behaviors, and general well-being. The COVID-19 survey was a measure that was developed and used by researchers at the University of North Carolina but has not been validated. The COVID-19 survey was sent separately from the regularly scheduled RECOVERY surveys, and participants were informed that additional remuneration was not available. The RECOVERY study and the additional COVID-19 survey were approved by the Boston Children’s Hospital Institutional Review Board.

### Survey measures

Sample demographic characteristics were obtained from the participants’ baseline RECOVERY surveys. The authors obtained permission to use survey questions on ED thoughts and behaviors due to COVID-19 that were developed and used by researchers at the University of North Carolina Center of Excellence for Eating Disorders [3]. Additional RECOVERY COVID-19 survey questions used in the following analyses, including those regarding treatment access, changes to care and disruptions in care, were developed by the authors.

#### Primary predictor variables

##### Access to care

Participants were asked about their access to multi-disciplinary ED care since the pandemic via the question, “I have been able to access my providers …” . Participants could then check all that applied: “via telehealth,” “in person,” “neither (I have not been able to access my providers at all).” Responses were then dichotomized to indicate any access to care (via telehealth or in-person) vs. no access.

##### Changes to care

Participants were asked about changes in different elements of their care in a series of two questions. First, respondents were asked to report what care they were receiving prior to the pandemic (i.e., “Before the pandemic, I was involved in …” ). Participants could then check all that applied from weight checks, nutrition, or therapy appointments. Participants were then asked to report on the care they have received since the pandemic (i.e., “Since the pandemic, I have been involved in …” ), and were asked to check all that applied from the same list of potential appointments. We then compared the pre- and post-pandemic reported care and created a variable indicating any stopped care (if any of the three —weight checks, therapy, or nutrition—changed from pre- to post-pandemic) v. no change to care.

##### Quality of care

Patients were asked to rate the overall quality of the care they received during the first few months of the pandemic, compared to care received prior to the pandemic, using a 4-point Likert scale: “better than usual”, “as good as usual”, “somewhat worse than usual,” and “worse than usual”.

##### Perceived treatment disruption

Patients were asked to report whether their overall ED treatment had been disrupted as a result of the COVID-19 pandemic with response options of yes, no, not applicable.

#### Primary outcomes

##### Intrusive ED thoughts and behaviors

Participants were asked, “how has the COVID-19 pandemic affected intrusive eating disorder thoughts” using a 5-point Likert scale, ranging from “increased significantly” to “decreased significantly.” Responses were dichotomized to indicate no increase vs. any increase in ED thoughts. Respondents also reported in three separate questions whether they had engaged in more restrictive behaviors, compensatory behaviors, or binging behaviors in the past three months “because of COVID-19 related factors.” Answers to these three questions were on a 4-point Likert scale ranging from “not at all” to “daily or more.” Responses from the three questions were combined and then dichotomized to indicate frequently or daily vs. never or rarely for any of the three ED behaviors.

#### Additional variables

##### Age

Age at time of COVID-19 survey completion was calculated from the date of survey completion and the date of birth obtained from the RECOVERY study baseline survey.

##### Sex

Self-reported sex assigned at birth (female, male or another sex) was obtained at the time of RECOVERY study baseline survey completion.

##### Race/ethnicity

Patients were asked to select all that applied from the following options on the RECOVERY baseline survey: Hispanic/Non-Hispanic, American Indian or Alaska Native, Asian, Black or African American, Middle Eastern/North African, Native Hawaiian or other Pacific Islander, White/Caucasian or another race. We constructed a mutually exclusive race/ethnicity variable consisting of non-Hispanic white, non-Hispanic Black or African-American, Asian, Multiracial, Other race, and Hispanic.

##### ED diagnosis

Patients self-reported their ED diagnosis by selecting all that applied from the following options on the RECOVERY baseline survey: anorexia nervosa (AN), atypical anorexia nervosa (AAN), Avoidant Restrictive Food Intake Disorder (ARFID), bulimia nervosa (BN), binge-eating disorder (BED), purging disorder, other eating issue(s)/disorder(s), and I don’t know/Unsure. AN and AAN were collapsed to create an indicator for anorexia nervosa vs. other ED diagnoses.

##### Length of treatment

This variable was calculated from the date of patient’s first ED clinic appointment to the date of COVID-19 survey completion.

### Statistical analysis

We examined frequencies (percent) for categorical variables and means (standard deviation) for continuous variables. For this subset analysis, RECOVERY participants who responded to the COVID-19 study were compared to participants in the RECOVERY study who did not respond to the COVID-19 survey on demographic factors (age, race/ethnicity and sex assigned at birth) and ED diagnosis using *t*-tests for continuous variables and *χ*^2^ tests for categorical variables. Multivariable logistic regression was used to examine the associations of access to, changes in, quality of, and perceived disruption to ED care during the COVID-19 pandemic with changes in ED thoughts and behaviors. Regression analyses were adjusted for age, sex at birth, race/ethnicity, ED diagnosis and length in treatment. All analyses were conducted using SAS (v9.4; Cary, NC).

## Results

### Sample characteristics

Of the 161 participants enrolled in the RECOVERY study, eighty-nine (55%) participants responded to the COVID-19 survey. Respondents to the survey did not differ from non-respondents on age at enrollment, race/ethnicity, sex assigned at birth. Participants with restrictive ED diagnoses (AN and AAN) were more likely to respond to the survey (*p* = 0.03) compared to those with non-restrictive diagnoses (BED, BN, ARFID, purging disorder, other eating issue(s)/disorder(s), or I don’t know/Unsure).

Of the eighty-nine respondents, sixteen (18%) were excluded from further analysis because they were not engaged in treatment prior to the pandemic. Of these remaining 73 participants, the mean age was 19.1 ± 3.0 years (Table 1). The majority were female (93%), White/non-Hispanic (79%) and reported a diagnosis of AN or AAN (85%). Approximately half (53%) had been in treatment for two or more years.

**Table 1:** Demographic characteristics, eating disorder diagnosis and report of ED symptomatology of RECOVERY participants who completed the COVID-19 survey (*N* = 73)

	Overall (*N* = 73)
**Age at survey completion** (years), *mean (SD)*	19.1 (3.0)
**Age over 18 at survey completion**	48 (66%)
**Female at birth**	68 (93%)
**Race/Ethnicity**	
White, non-Hispanic	58 (79%)
Asian	5 (7%)
Hispanic	4 (6%)
Black	1 (1%)
Multiracial	4 (6%)
Other race	1 (1%)
**Restrictive Eating Disorder Diagnosis**	62 (85%)
**Length of ED Treatment**	
< 1 year	8 (11%)
1–2 years	26 (36%)
2 years or more	39 (53%)
**Intrusive ED thoughts as a result of COVID-19**	59 (81%)
**ED Behaviors as a result of COVID-19**	59 (81%)

### Access to, changes in, quality of, and perceived disruptions to care

Access to care remained high, with 92% of respondents reporting continued access to at least one provider via telehealth or in person (Table 2). Of those continuing in care, 88% used telehealth to see at least one provider. However, nearly half (47%) of the 73 participants who were actively engaged in ED care prior to the pandemic reported stopping at least one aspect of their ED treatment: sixteen (22%) stopped mental health counseling/therapy, seven (10%) stopped nutrition visits, and twenty-three (32%) stopped weight checks with their medical provider. Approximately one-third (32%) perceived a disruption in treatment. Of those with telehealth access (*n* = 64), 9% found care to be better than usual, 59% as good as usual, while 30% said somewhat worse than usual, and 2% much worse. There was no association between access to care via telehealth and perceived disruption of care (*p* = 0.99) or quality of care (*p* = 0.36), however respondents who perceived a disruption to their treatment were more likely to report lower quality of care (*p* = 0.004).

**Table 2:** Markers of care and unadjusted association with ED thoughts and behaviors during the COVID-19 pandemic (*N* = 73)

	Overall (*N* = 73) N (%)	Intrusive ED Thoughts			ED Behaviors^a^		
		n (%)		***p***-value	n (%)		***p***-value
		Increased (*n* = 59)	No change/ decreased (*n* = 14)		Frequently or daily (*n* = 33)	Rarely or Never (*n* = 40)	
***Access***							
**Accessed via telehealth**^†^	64 (88%)	53 (90%)	11 (79%)	0.36	29 (88%)	35 (88%)	0.99
**Accessed in-person**^†^	16 (22%)	13 (22%)	3 (21%)	0.99	7 (21%)	9 (23%)	0.89
**No access**	6 (8%)	3 (5%)	3 (21%)	0.08	3 (9%)	3 (8%)	0.99
**Any access to care**				0.08			0.99
Yes (telehealth and/or in-person)	67 (92%)	56 (95%)	11 (79%)		30 (91%)	37 (92%)	
No	6 (8%)	3 (5%)	3 (21%)		3 (9%)	3 (8%)	
***Change in therapy***							
**Outpatient therapy**				0.49			0.66
No change	57 (78%)	47 (80%)	10 (71%)		25 (76%)	32 (80%)	
Stopped during pandemic	16 (22%)	12 (20%)	4 (29%)		8 (24%)	8 (20%)	
**Outpatient nutrition**				0.99			0.99
No change	66 (90%)	53 (90%)	13 (93%)		30 (91%)	36 (90%)	
Stopped during pandemic	7 (10%)	6 (10%)	1 (7%)		3 (9%)	4 (10%)	
**Weight checks by medical provider**				0.12			0.22
No change	50 (68%)	43 (73%)	7 (50%)		25 (76%)	25 (63%)	
Stopped during to pandemic	23 (32%)	16 (27%)	7 (50%)		8 (24%)	15 (38%)	
**Stopped any treatment**				0.38			0.44
Yes	34 (47%)	26 (44%)	8 (57%)		16 (48%)	23 (58%)	
No	39 (53%)	33 (56%)	6 (43%)		17 (52%)	17 (42%)	
***Disruption***							
**Perceived disruption to care**				0.36			0.56
Yes	24 (32%)	21 (36%)	3 (21%)		12 (36%)	12 (30%)	
No	49 (67%)	38 (64%)	11 (79%)		21 (64%)	28 (70%)	
***Quality***							
**Quality of treatment in past 3 months**				**0.01**			0.51
Better than usual	6 (8%)	2 (3%)	4 (31%)		1 (3%)	5 (13%)	
As good as usual	42 (59%)	34 (59%)	8 (62%)		19 (59%)	23 (59%)	
Somewhat worse than usual	21 (30%)	20 (34%)	1 (8%)		11 (34%)	10 (26%)	
Much worse than usual	2 (3%)	2 (3%)	0 (0%)		1 (3%)	1 (3%)	

† not mutually exclusive – *n* = 13 reported access both via telehealth and in-person

a Composite measure for engaging in restrictive or compensatory behaviors or binging on food in the past 3 months

### ED thoughts and behaviors

Many respondents (81%) endorsed increased intrusive ED thoughts and behaviors as a result of the COVID-19 pandemic. Thirty-three (45%) participants reported engaging in restrictive/compensatory/or binging behaviors frequently or daily.

### The association of access to, changes in, perceived quality of and perceived disruption to care with ED thoughts and behaviors

Unadjusted and adjusted associations between i) access to, ii) changes in, iii) perceived quality of and iv) perceived disruption to care, with ED thoughts and behaviors are presented in Table 3. After adjusting for age, sex assigned at birth, race/ethnicity, restrictive ED diagnosis and length of treatment, there were no significant associations between our markers of care described and ED thoughts or behaviors. However, those who perceived treatment disruption had higher odds of intrusive ED thoughts (adjusted odds ratio = 2.63; 95% CI: 0.56–12.3) and increased ED behaviors (aOR = 1.98; 95% CI: 0.63–6.19). Those who still had access to ED care had higher odds of intrusive ED thoughts (aOR = 5.32; 95% CI: 0.72–39.6) but lower odds of increased ED behaviors (aOR = 0.67; 95% CI: 0.11–4.20). Patients who stopped some treatment had lower odds of intrusive ED thoughts (aOR = 0.76; 95% CI: 0.20–2.88), but higher odds of increased ED behaviors (aOR = 2.05; 95% CI: 0.70–6.05). Finally, we observed those who perceived quality of care as worse than usual were more likely to have intrusive ED thoughts (aOR = 8.00; 95% CI: 0.85–75.7), though these associations were approaching significance (*p* = 0.07).

**Table 3:** Unadjusted and adjusted odds of ED thoughts or behaviors associated with markers of care (*N* = 73). Model adjusts for age, sex at birth, race/ethnicity, restrictive diagnosis and length of treatment

Predictor^a^	Outcome							
	Intrusive ED Thoughts^b^				ED Behaviors^c^			
	Unadjusted		Adjusted^d^		Unadjusted		Adjusted^d^	
	OR (95% CI)	*p*-value	OR (95% CI)	*p*-value	OR (95% CI)	*p*-value	OR (95% CI)	*p*-value
**Perceived treatment disruption**		0.32		0.22		0.57		0.24
Yes	2.03 (0.51, 8.08)		2.63 (0.56, 12.3)		1.33 (0.50, 3.55)		1.98 (0.63, 6.19)	
No	1.0 (Ref.)		1.0 (Ref.)		1.0 (Ref.)		1.0 (Ref.)	
**Any access to care**		0.06		0.11		0.81		0.67
Yes	5.09 (0.91, 28.6)		5.32 (0.72, 39.6)		0.81 (0.15, 4.31)		0.67 (0.11, 4.20)	
No	1.0 (Ref.)		1.0 (Ref.)		1.0 (Ref.)		1.0 (Ref.)	
**Stopped any treatment**		0.38		0.68		0.44		0.19
Yes	0.59 (0.18, 1.92)		0.76 (0.20, 2.88)		1.44 (0.57, 3.63)		2.05 (0.70, 6.05)	
No	1.0 (Ref.)		1.0 (Ref.)		1.0 (Ref.)		1.0 (Ref.)	
**Quality of treatment**		0.06		0.07		0.41		0.37
As good or better than usual	1.0 (Ref.)		1.0 (Ref.)		1.0 (Ref.)		1.0 (Ref.)	
Somewhat or much worse than usual	7.33 (0.89, 60.3)		8.00 (0.85, 75.7)		1.53 (0.56, 4.15)		1.65 (0.55, 4.88)	

*Abbreviations*: *OR* odds ratio, *CI* confidence interval

a Separate models predicting outcome from each predictor

b Intrusive ED thoughts increased vs. no change/decreased

c Composite measure for engaging in restrictive or compensatory behaviors or binging on food frequently or daily vs. never or rarely in the past 3 months

d Models adjusting for age, sex at birth, race/ethnicity, restrictive diagnosis and length of treatment

## Discussion

This study examined the association between markers of care and ED thoughts and behaviors in youth, five months after Massachusetts enacted stay-at-home orders due to the COVID-19 pandemic. Our participants reported marked increases in both intrusive ED thoughts and behaviors, which they attributed to the COVID-19 pandemic. Reassuringly, the majority of our cohort was able to maintain access to at least one member of their care team via telehealth and some were able to continue to see their providers in person. However, half had to stop some aspect of their treatment because of the pandemic. Despite these changes, patient-reported quality of care did not suffer, and most patients did not perceive a disruption to their care. Those who perceived a treatment disruption to care were more likely to rate the quality of their care lower than those who did not perceive a disruption. Our results did not support our hypothesis as none of the markers of care described was statistically associated with increased ED thoughts and behaviors.

Like many institutions across the globe [11, 18–20], our clinicians transitioned their practices to videoconferencing platforms shortly after stay-at-home advisories were issued in March 2020 [21]. Despite providers’ concerns [12–14, 22], the majority of our patients who accessed their care via telehealth felt their quality of care was as good as, or better, than usual. Previous research has shown telehealth to be effective for use in psychotherapy [21, 23], as well as effective in adolescent/young adult populations [24–26], however little research exists examining the use of this technology for multi-disciplinary ED treatment [27, 28]. Our results suggest providing ED care via telehealth is well accepted and provides good quality of care for ED patients. Although more research is necessary to determine efficacy [4, 29], these findings serve as preliminary evidence for the use of multidisciplinary ED care via telehealth. Moreover, the expansion and continued use of telehealth for multi-disciplinary ED treatment could mitigate barriers to care and increase access for patients who are less connected or away for college or employment, even after the restrictions of the pandemic are lifted [30]. Similar to other studies, our patients with EDs reported increases in ED thoughts and behaviors related to the COVID-19 pandemic [3, 5, 31, 32]. Although our multivariate analyses were not statistically significant, the association of continued care with increased ED thoughts but lower ED behaviors was not expected. The relationship between sustained ED treatment and increased ED cognitions may be confounded by the COVID-19 restrictions and isolation from peers, which may have contributed to increased ED thoughts. One study attributed an increase in ED thoughts during the COVID-19 pandemic to the lack of in-person care and absence of distractions [33]. Our findings also suggest that continued access to treatment may serve as a protective factor, preventing an increase in ED behaviors. This finding is comparable to another study by Schlegl and colleagues, who found an increase in ED cognitions, but not an increase in behaviors during the initial months of the COVID-19 pandemic [5]. Stay-at-home orders due to COVID-19 may have led to increased parental presence and involvement, which could have also contributed to lower ED behaviors. Ultimately, it is critical to understand how to support our patients so that they may remain resilient against their ED behaviors, particularly during times of stress and isolation, which are known risk factors for patients with EDs [32–36].

### Limitations

Our study was underpowered due to the moderate response rate to the COVID-19 survey and the high prevalence of ED symptomatology in our sample. Post-hoc power calculations coupled with wide confidence intervals suggest that we were underpowered to detect statistically significant associations between our markers of care and ED symptomatology. Given the effect size we observed for the association between access to care and intrusive ED thoughts, we had 66% power to detect a statistically significant difference. Our findings were limited by the response rate; just over half of participants in the RECOVERY study responded to the COVID-19 survey. There is a possibility that those who did not respond were affected by COVID-19 differently than those who did respond, which could affect the results of this study. Notably, this survey was sent out of sequence and remuneration was not available for completion of this survey, which may explain some loss of respondents. Overall, there were no differences between survey respondents and non-respondents in age, race/ethnicity, sex, but those with restrictive EDs were more likely to respond to the survey.

An additional limitation is the generalizability of our findings. The majority of our study participants identified as White and were diagnosed with restrictive EDs. Additionally, our participants may be more representative of individuals who were already engaged in care and connected to their health care team, as most participants were in treatment and the majority had been in treatment for more than two years. All of the participants in this cohort had providers with ED expertise at some point, making it an easier transition to COVID-19 modified treatment. Published literature has reported increased ED incidence as a result of the pandemic [37], which is consistent with our clinical experience during this time. Anecdotally, we have noted that it is especially difficult for these new patients to find providers. Thus, our findings may not be generalizable to patients with new onset EDs.

### Future studies

Future studies should examine the effect of COVID-19 on the incidence of EDs and access to care for new patients. Given our findings showing no differences in patient perception of quality of care via telehealth versus in person care, future research could explore associated factors that may contribute to the acceptability of telehealth for multidisciplinary ED treatment and investigate objective ED outcomes of patients seen via telehealth versus in-person. Finally, further study is needed to discern the differences in markers of care by ED diagnosis and demographic groups, particularly with respect to race/ethnicity and socioeconomic status.

## Conclusions

The present study demonstrates the profound impact COVID-19 has had on patients’ ED symptomatology and access to treatment. Our findings suggest that patients with established care teams prior to the pandemic were able to maintain some aspect of their care via telehealth and perceived it to be high quality. Our patients with EDs reported marked increases in intrusive ED thoughts and behaviors because of the pandemic. Disruption in, decreased quality of, changes to and decreased access to care were not statistically significantly associated with increased ED symptomatology, though this was likely due to lack of statistical power. More work is needed to better understand the relationship between care changes during the pandemic and ED symptomatology in larger populations. In the meantime, providers should continue to support patients via telehealth, and in person when possible, who are at risk of worsening EDs during this difficult time.

## Data Availability

The datasets generated and/or analyzed during the current study are not publicly available due protection of patient privacy but are available de-identified from the corresponding author on reasonable request and IRB approval.

## Abbreviations

ED: Eating disorder
AN: Anorexia nervosa
AYA: Adolescents’/young adults’
RECOVERY: Registry of Eating Disorders and their Co-Morbidities Over Time in Youth
AAN: Atypical anorexia nervosa
ARFID: Avoidant Restrictive Food Intake Disorder
BN: Bulimia nervosa
BED: Binge-eating disorder
